# Supplementary material for: Unraveling the transcriptional features and gene expression networks of pathogenic and saprotrophic Ophiostoma species during the infection of Ulmus americana
Source: Microbiol Spectr. 2024 Jan 17;12(2):e03694-23. doi: 10.1128/spectrum.03694-23 (PMC10845970; doi:10.1128/spectrum.03694-23)
Supplement: Supplemental Figures S1 to S4 — Supplementary figures. [file spectrum.03694-23-s0001.pdf]

Unraveling the transcriptional features and gene expression networks of pathogenic and saprotrophic *Ophiostoma* species during the infection of *Ulmus americana*

Thais C. de Oliveira[1,2*], Nastasia J. Freyria[3], Jorge Luis Sarmiento-Villamil[1,2,4], Ilga Porth[1,2], Philippe Tanguay[5] and Louis Bernier[1,2*]

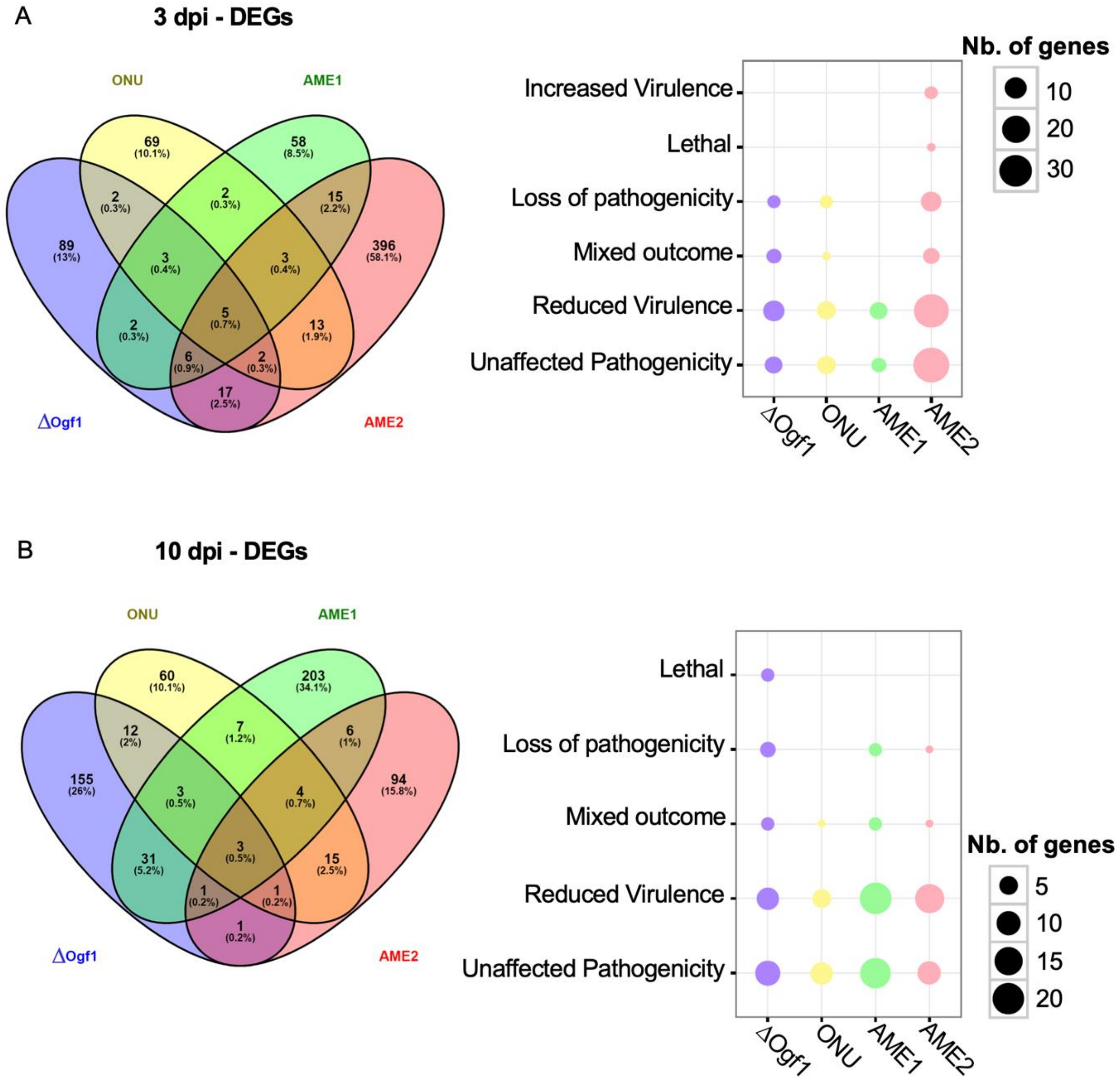

Supplementary Figure 1. Venn diagrams of ONU, ΔOgf1, AME1 and AME2 DEGs identified at 3 dpi (A) and 10 dpi (B), followed by a comparison of DEGs with orthologs in PHI-base that appeared exclusively in each organism.

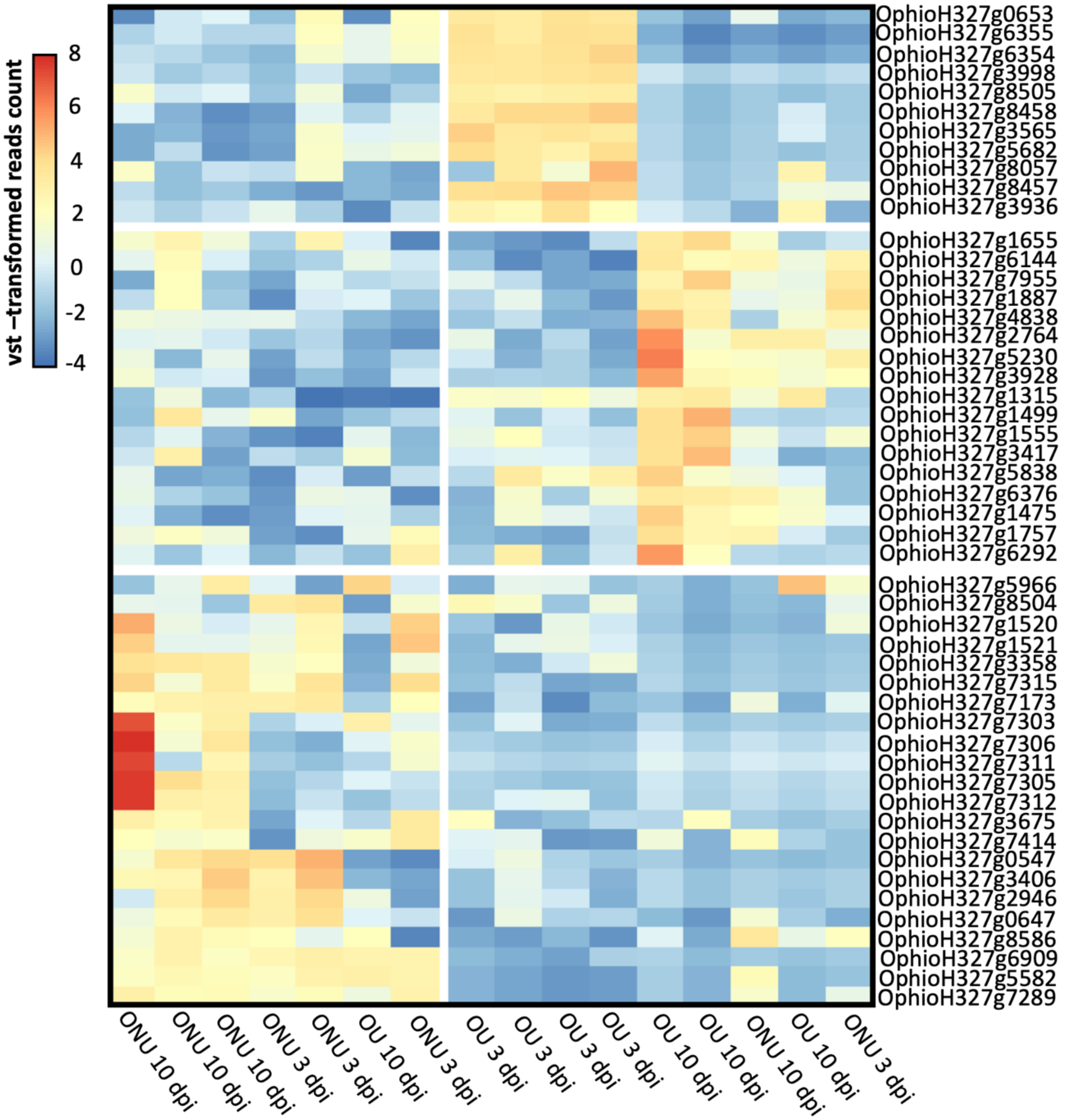

Supplementary Figure 2. Heatmap of clustered results based on normalized and transformed read counts showing the top 50 genes with the most variant expression (vst) among OU vs ONU biological replicates. Rows and columns were clustered and expression amount was plotted in $\log_2$ scale using the *pheatmap* package in R.

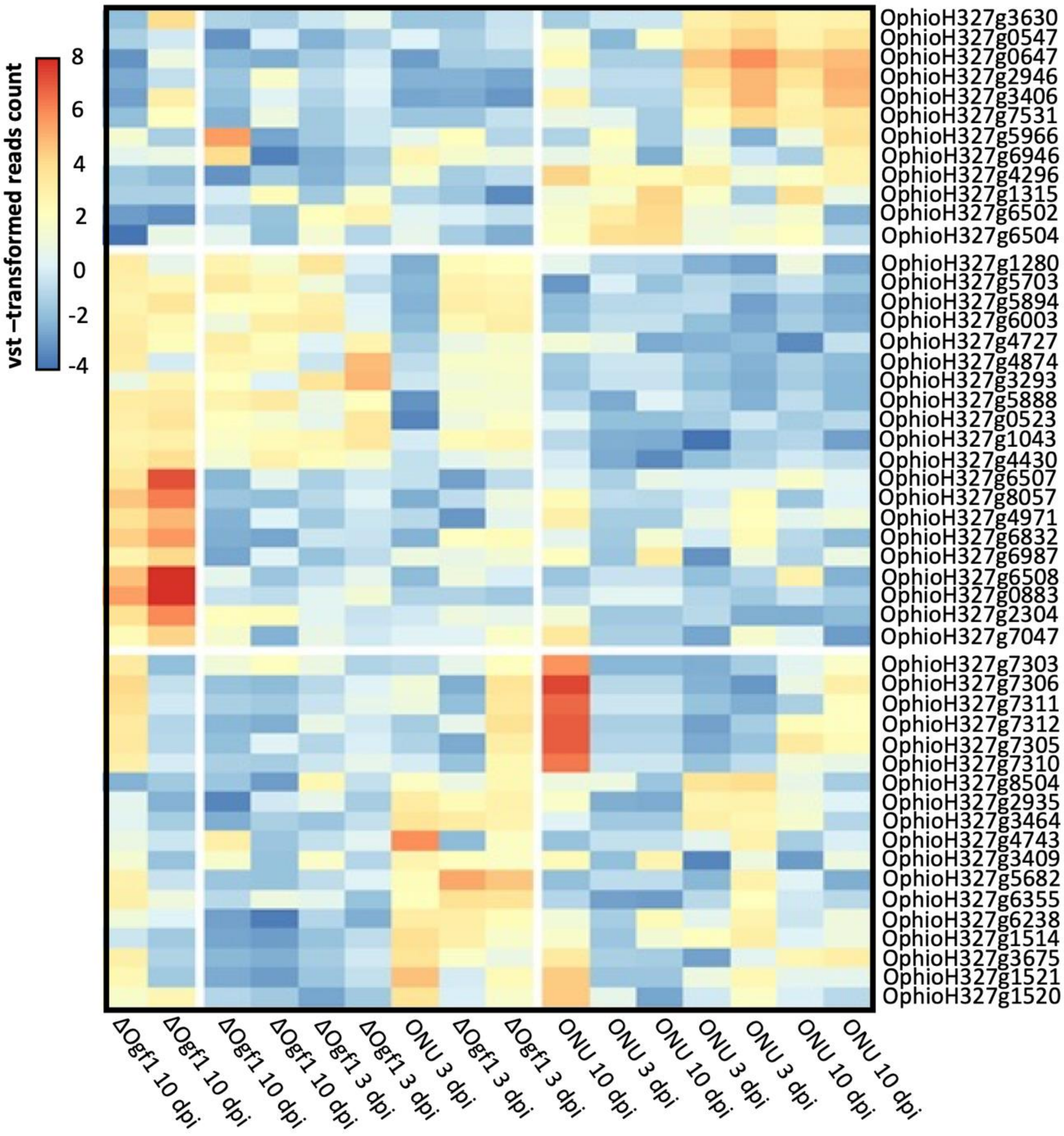

Supplementary Figure 3. Heatmap of clustered results based on normalized and transformed read counts showing the top 50 genes with the most variant expression (vst) among ONU vs ΔOgf1 biological replicates. Rows and columns were clustered and expression amount was plotted in $\log_2$ scale using the *pheatmap* package in R.

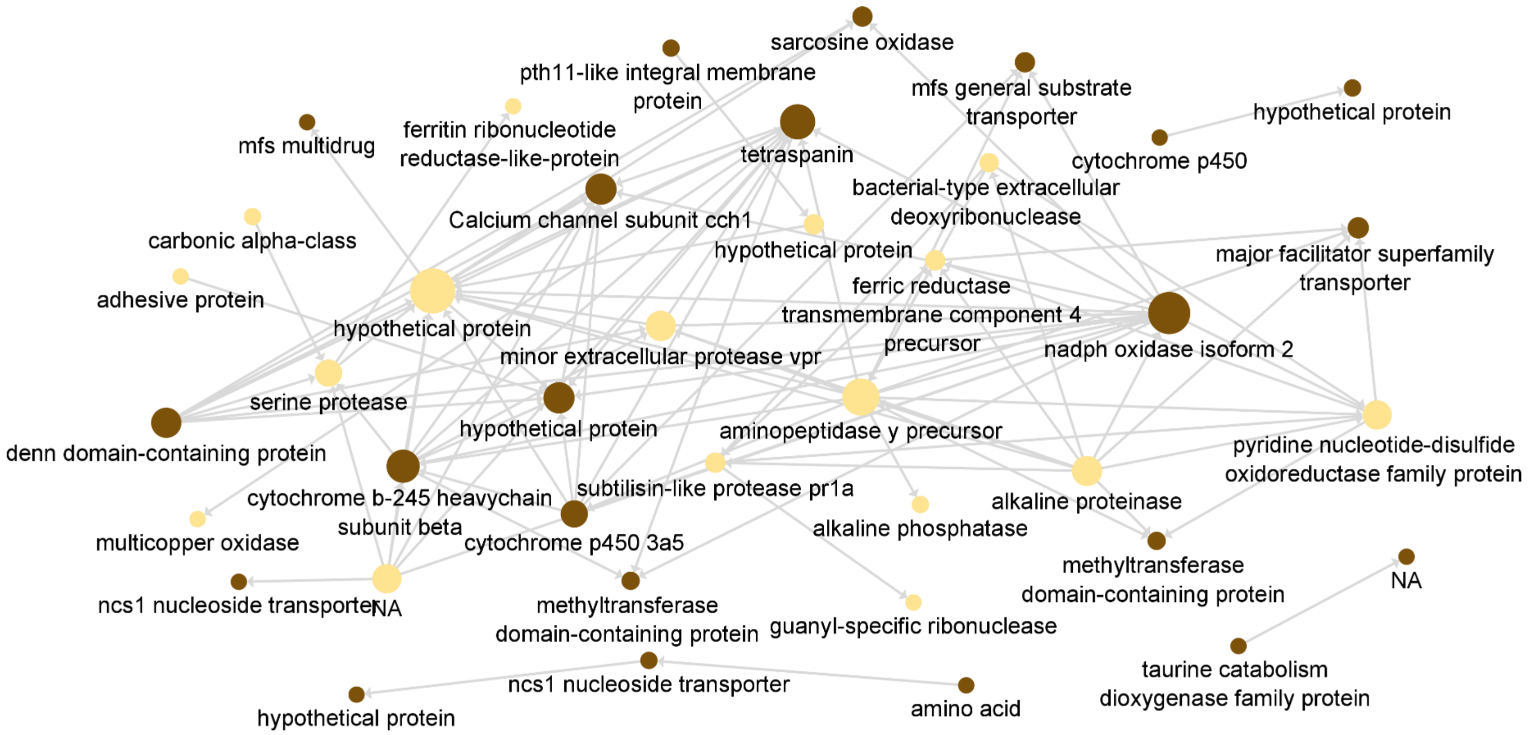

Supplementary Figure 4. Partial co-expression network for the Brown module based on WGCNA analysis. The brown module was highly correlated with 3 dpi expression data ($p < 0.01$). The pale-yellow color represents genes encoding proteins with a peptide signal. The node size is proportional to the number of links with other genes in the module.
